# Clinical Characteristics, Antimicrobial Resistance, and Outcomes of Patients with Invasive Pneumococcal Disease in Ningxia Hui Autonomous Region, China, 2013–2021

**DOI:** 10.1155/2022/1262884

**Published:** 2022-12-12

**Authors:** Jia Tao, Wen Wang, Gang Li, Linlin Wang, Liang Wang, Zhijun Zhao, Wei Jia, Liru Wang

**Affiliations:** ^1^Center of Medical Laboratory, General Hospital of Ningxia Medical University, Yinchuan 750004, China; ^2^Institute of Medical Sciences, General Hospital of Ningxia Medical University, Yinchuan 750004, China; ^3^Ningxia Key Laboratory of Clinical and Pathogenic Microbiology, General Hospital of Ningxia Medical University, Yinchuan 750004, China

## Abstract

**Objectives:**

This study aimed to analyze the clinical features, antibiotic susceptibility profiles, and outcomes of patients with invasive pneumococcal disease (IPD) at a hospital in Ningxia Hui Autonomous Region, to provide the basis for improving the clinical treatment effect.

**Methods:**

Patients with IPD were retrospectively collected from 2013 to 2021. Clinical manifestations, laboratory tests, antimicrobial susceptibility, antibiotic treatment, and outcomes of the disease were analyzed.

**Results:**

In this study, we identified 127 IPD cases, of whom 49 (38.6%) had meningitis and 78 (61.4%) had bacteremia. The median ages of pediatric cases and adult cases were 2 years (IQR: 0–5) and 52.5 years (IQR: 35–62), respectively. There were 27 and 45 males in the pediatric and adult groups, and no significant gender difference in the different age groups (*p* = 0.584) was found. Of 75 cases with underlying diseases, pneumonia (11%), malignancy (11%), hypertension (9.4%), and hepatic cirrhosis (7.9%) were the most common. The incidence of underlying diseases was even higher in the adult group (67.1%) than in the pediatric group (47.1%) (*p* = 0.028). The frequency of fever, cough, and seizures was significantly higher in the pediatric group than in the adult group, with *p*-values of 0.004, 0.004, and 0.001, respectively. The percentage of neutrophils in the blood was significantly higher in the adult cases than in the pediatric cases (*p*  <  0.001). Furthermore, there was a significantly higher WBC count (*p*  <  0.001), percentage of neutrophils (*p* = 0.012), and protein level (*p* = 0.019) in the CSF samples in the adult patients compared to pediatric patients. The susceptibility rates of *S. pneumoniae* isolates to vancomycin, linezolid, and levofloxacin were 100%. The susceptibility rates of penicillin were 98.7% and 34.1% in bacteremia and meningitis patients, respectively. Most isolates were resistant to erythromycin, clindamycin, tetracycline, and azithromycin. The most common antibiotic treatment was *β*-lactams. Seven (5.5%) patients died during hospitalization, and 38 (29.9%) patients' health deteriorated.

**Conclusion:**

These results may provide a reference basis for the diagnosis and empiric treatment of IPD in the region.

## 1. Background

*Streptococcus pneumoniae (S. pneumoniae)* is a Gram-positive opportunistic pathogen that colonizes the upper respiratory tract of humans. *S. pneumoniae* is carried by up to 27%–65% of children and 10% of adults [1, 2]. This asymptomatic colonization can progress to a broad spectrum of diseases ranging from mild noninvasive disease to life-threatening invasive pneumococcal disease (IPD) [3, 4]. IPD refers to the isolation of *S. pneumoniae* from normally sterile sites such as blood and cerebrospinal fluid [5]. The most severe form of IPD mainly includes meningitis and bacteremia. Infected people were mainly children, the elderly, and people with various underlying illnesses. Pneumococcal meningitis and bacteremia are important causes of high morbidity and mortality in the world [6]. Coronas et al. reported that the IPD incidence ranged from 2.4 to 3.0/100,000 in children under 18 years of age and from 9.5 to 15.9/100,000 in children under 2 years of age [7]. Meanwhile, it has been reported that IPD has a high mortality rate from 9.6% for those aged 17–54 years to 31.7% for those aged ≥ 75 years [8]. The Global Invasive Bacterial Vaccine-Preventable Diseases (IB-VPD) Surveillance Network group reported that the case fatality ratio of pneumococcal meningitis was 12.2% for children < 5 years of age [9]. An eleven years study in India showed that the overall fatality rate of IPD was 17.8%, and 24.3% and 18.4% for meningitis and septicemia, respectively [10]. In 2015, there were estimated to be 83,900 cases and 37,900 deaths in children caused by pneumococcal meningitis worldwide [11].

An analysis showed that prognostic factors for mortality in IPD in adults were age, nosocomial infection, septic shock, underlying chronic diseases, solid organ tumors, immunosuppressed status, and alcohol abuse [12]. Despite the wide adoption of pneumococcal conjugate vaccination reducing the incidence of IPD, the overall mortality rate from IPD has remained high, and the antibiotic resistance in *S. pneumoniae* is increasing [13]. A study conducted in different cities in northern China reported the percentage of penicillin-resistant* S. pneumonia* (PRSP) from invasive pneumococcal isolates was 56.7% [14]. A multicenter study from China revealed that 67.7% of the isolates were classified as PRSP based on meningitis (*R* ≥ 0.12 *μ*g/ml) breakpoint [15]. A study of children with IPD in Beijing, China between 2012 and 2017 showed the nonsusceptibility rates of penicillin among nonmeningitis patients increased from 31.3% in 2012 to 68.2% in 2017, and the nonsusceptibility rates of meningitis isolate fluctuated by year [16]. These findings suggest that the antibiotic resistance of *S. pneumoniae* in China is a serious public health problem.

However, there are few studies reported the clinical characteristics of patients with meningitis and bacteremia to *S. pneumoniae*, and the antibiotic resistance of *S. pneumoniae* in Ningxia Hui Autonomous Region (NHAR), which is a small landlocked autonomous region in northwestern China. The purpose of this study was to investigate the clinical characteristics, the outcomes of patients with pneumococcal meningitis and bacteremia, and antibiotic resistance of *S. pneumonia* isolates at a teaching hospital in NHAR.

## 2. Materials and Methods

### 2.1. Patients and Definition

The clinical records of all patients with positive blood and/or cerebral spinal fluid (CSF) cultures for *S. pneumoniae* at the General Hospital of Ningxia Medical University in NHAR from January 2013 to December 2021 were reviewed retrospectively. Isolate from both blood and CSF was classified as meningitis. This study was approved by the ethics committee of the General Hospital of Ningxia Medical University.

### 2.2. Data Collection

The medical records were reviewed to collect the data of patients. The study data included the following variables: demographic characteristics (age and gender), underlying diseases, clinical symptoms, length of stay in the hospital, antimicrobial treatment, and outcome. Routine laboratory tests such as peripheral white blood cell (WBC) count, neutrophil percentage, and procalcitonin (PCT) were collected. CSF WBC count, glucose level, total protein content, and chloride ion (Cl^−^) level were recorded.

### 2.3. Antibiotic Susceptibility Testing

Blood or CSF samples were cultured using BACT/ALERT 3D blood culture system (bioMérieux, USA). *S. pneumoniae* isolates were identified by typical colony morphology in blood agar, optochin (Oxoid Company, Britain) susceptibility, and Vitek MS system (bioMérieux, USA). For patients with multiple isolates, only the first isolate was used to test antibiotic susceptibility. Minimum inhibitory concentrations (MIC) of penicillin (P), ceftriaxone (CRO), cefotaxime (CTX), and cefuroxime (CXM) were determined using Vitek 2 compact system (bioMérieux, USA) or E-test (Oxoid Company, Britain). Susceptibility testing of clindamycin (DA), azithromycin (AZM), erythromycin (E), linezolid (LZD), vancomycin (VA), chloramphenicol (C), levofloxacin (LEV), trimethoprim/sulfamethoxazole (SXT), and tetracycline (TE) were performed by the Vitek 2 compact system or disk diffusion method (Oxoid Company, Britain). The results of antimicrobial susceptibility testing were interpreted according to the CLSI (2020) guideline [17].

### 2.4. Statistical Analysis

Categorical variables were presented as the number of cases and percentages and compared using Fisher's exact test. Continuous variables that did not follow a normal distribution were described as median with 25th and 75th percentiles (IQR, 25th-75th percentile), and compared using the Mann–Whitney *U* test. Statistical analysis was performed using GraphPad Prism software version 8 (GraphPad Software Inc., San Jose, CA, USA); a two-tailed *p* value <0.05 was considered to be statistically significant. Antimicrobial susceptibility test results were analyzed using WHONET 5.6 software.

## 3. Results

### 3.1. Demographic and Clinical Characteristics of the Patients

**Figure 1:**
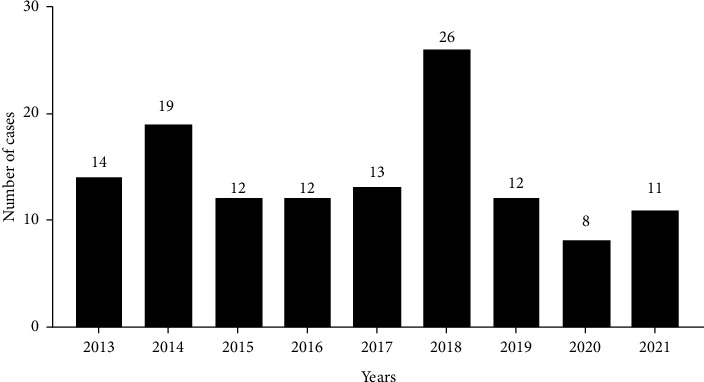
Number of patients with IPD in Ningxia Hui Autonomous Region, China, 2013–2021.

**Figure 2:**
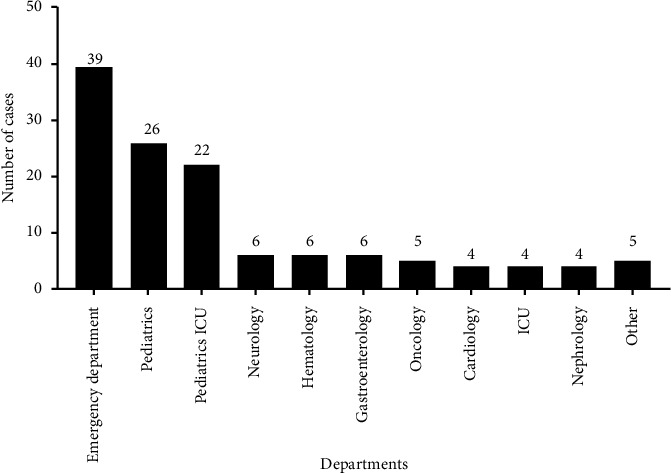
Departments distribution of patients with IPD in Ningxia Hui Autonomous Region, China, 2013–2021.

During the period of study, 127 *S. pneumoniae* invasive infection episodes were finally included. Data about the distribution of cases per year of study are shown in Figure 1. Enrolled patients included 49 cases of meningitis and 78 of bacteremia. The number of pediatric (≤16 years old) and adult (>16 years old) patients was 51 and 76, respectively. The median ages of pediatric cases and adult cases were 2 years (IQR: 0–5) and 52.5 years (IQR: 35–62), respectively. There were 27 and 45 males in the pediatric and adult groups, and no significant gender difference in the different age groups (*p* = 0.584) was found. Patients in this study were mainly concentrated in the emergency department (39), pediatrics (26), and pediatric ICU (22) (Figure 2). Among the overall population, 75 patients had underlying diseases, and 15 of them had at least two kinds of diseases. The primary underlying diseases were pneumoniae (*n* = 14) and malignancy (*n* = 14), other diseases mainly including hypertension (*n* = 12), hepatic cirrhosis (*n* = 10), anemia (*n* = 8), brain trauma (*n* = 8), nephrotic syndrome (*n* = 5), heart disease (*n* = 3), rheumatoid arthritis (*n* = 2), renal failure (*n* = 2), and hepatitis (*n* = 2). There was one patient that had Bucca's syndrome, liver failure, ileus, primary immunodeficiency, acute tonsillitis, umbilical hernia1, and hydrocephalus recurrence, respectively. Out of the 75 patients with underlying diseases, 24 were pediatric patients, and the remaining were adults. There was a significant difference in the proportion of underlying diseases between the two groups (*p* = 0.028). The existence of pneumonia was significantly higher in pediatric patients (*p* = 0.019). In contrast, cirrhosis and hypertension were more commonly identified in adult patients, with *p* values of 0.006 and 0.002, respectively. Twenty patients received mechanical ventilation and 74 patients received oxygen. The most common symptom was fever which was presented in 81.9% (104/127) of patients, followed by cough (26.8%, 34/127), vomiting (19.7%, 25/127), consciousness obstacle (18.9%, 24/127), headache (17.3%, 22/127), sputum (15.0%, 19/127), seizures (11.8%, 15/127), abdominal pain (7.1%, 9/127), hematemesis (3.9%, 5/127), breathing difficulty (3.9%, 5/127), hematochezia (3.2%, 4/127), other symptoms including chest pain, hemoptysis, sore throat. Then, we compared the symptoms at presentation between the two groups. The frequency of fever (*p* = 0.004), cough (*p* = 0.004), and seizures (*p* = 0.001) was significantly higher in the pediatric group than in the adult group. However, there was no significant difference between the groups concerning other symptoms, including vomiting (*p* = 0.255), consciousness obstacle (*p* = 0.821), headache (*p* = 0.094), and sputum (*p* = 0.805).  Table 1 summarized the comparison of demographic and clinical characteristics of the study population.

### 3.2. Laboratory Data

Hematic parameters, PCT testing, and CSF analysis were measured in 125, 96, and 49 patients, respectively. The median of WBC count was 13.7 (IQR: 3.5–24.0) *∗* 10^9^/L, and the median of neutrophil percentage was 74.9% (IQR: 59.3%–90.5%). Elevated WBC count was present in 60% (30/51) pediatric patients and 52% (39/76) adult patients, and there was no significant difference between the two groups (*p*=0.463). However, the percentage of patients with increasing neutrophil percentage was higher in adult patients (60/76) than in pediatric patients (23/51) (80% vs 46%, *p*  <  0.001). PCT testing results showed that 19 results were in the normal range (PCT < 0.5 ng/ml), 77 were increased (23, 0.5 ≤ PCT < 2 ng/ml; 19, 2 ≤ PCT < 10 ng/ml; 35, PCT ≥ 10 ng/ml). There was no significant difference in PCT testing results between the two groups (*p*=0.998). There was a significantly higher WBC count (*p*  <  0.001), percentage of neutrophils (*p*=0.012), and protein level (*p*=0.019) in CSF samples in the adult patients compared to pediatric patients. Additionally, there was no difference between glucose, and cl^−^ levels in CSF samples between the two groups.  Table 2 summarized the comparison of laboratory analysis of patients.

### 3.3. Antibiotics Susceptibility Testing

*S. pneumoniae* was isolated from blood alone in 78 patients, CSF alone in 36 patients, and both blood and CSF in 13 patients. Antimicrobial susceptibility testing results are shown in Table 3. According to the breakpoint of CLSI guideline, no isolate was resistant to vancomycin, linezolid, and levofloxacin with 100% susceptibility rates. The susceptibility rates of penicillin were 98.7% and 34.1% in bacteremia patients and meningitis patients, respectively. Meanwhile, 91.9% and 59.1% of isolates were sensitive to ceftriaxone in bacteremia and meningitis patients, and 87.5% and 50% to cefotaxime, respectively. Most isolates (more than 85%) were resistant to erythromycin, clindamycin, tetracycline, and azithromycin.

### 3.4. Antimicrobial Therapy and Outcome

Of forty-nine patients with meningitis, 47 (95.9%) received initiated antibiotic treatment before the isolates were identified. The most common antibiotic was *β*-lactams, which was used for 46 (97.8%) patients. There were 20 (42.5%) patients who were treated with two kinds of antibiotics, the most common pattern is a combination of *β*-lactams and vancomycin. Thirty-one patients had changed antibiotics according to the susceptibility of the pathogen. Among these patients, 19 (61.29%) were treated with vancomycin in a combination with ceftriaxone or meropenem. One patient received vancomycin in combination with ceftazidime. Four patients had vancomycin or ceftriaxone alone, respectively. Of 78 patients with bacteremia, 76 (97.44%) received empiric treatment, and the antibiotics mainly included *β*-lactams, vancomycin, and teicoplanin. Among them, 34 patients were given the same antibiotics after the antimicrobial susceptibility test. Forty-three patients had changed antibiotics according to the susceptibility of the pathogen. Twenty-three patients were treated with two kinds of antibiotics, the most common pattern is a combination of third-generation cephalosporin or carbapenems in a combination with vancomycin or teicoplanin. Of all the 127 cases, 82 (65%) patients were treated, and 38 (29.9%) deteriorated. The main reason for the deterioration of the disease was that patients gave up treatment. Seven patients died in the hospital, and the total mortality rate was 5.5%. The mortality rates of bacteremia and meningitis were 6.4% and 4.08%, respectively.

## 4. Discussion

Despite the prevention effectiveness of pneumococcal conjugate vaccines having been reported in more than 50 countries [18], the high prevalence of IPD and the increasing rates of penicillin and other antibiotic resistance are still global problems. Of note, clinical presentation and antimicrobial susceptibility patterns of *S. pneumoniae* vary geographically. In this study, we described the clinical characteristics and treatment outcomes of patients with *S. pneumonia* bacteremia and meningitis and examined the antimicrobial resistance of *S. pneumonia* isolates from 2013 to 2021 in a teaching hospital in the northwestern region of China. Our results showed males with pneumococcal bacteremia and meningitis had a higher occurrence than females, with a male/female ratio of 1.5 : 1, whereas there was no significant difference among gender. This result is consistent with a previous study [19]. However, Geng et al. reported that there were significantly more male patients than female patients [20]. In this study, the median age of all patients was 31 years and the percent of patients between <16 years and 16–60 years groups are similar (40.16% vs 40.94%), while the percentage of patients older than 60 years old is low (18.9%). Many previous studies have described a higher level of pneumococcal disease among adult patients over the age of 65 and children younger than 5 years old [21–23]. This could be explained by the fact that our hospital is a comprehensive teaching hospital that houses various specialist wards to accept more adults with underlying diseases. When we compared the subgroup in group <16 years old, we found 38 (74.5%) patients were younger than 5 years old. The minimum age of patients in this study is 1 day, however other studies reported the minimum age was 60 days or 6 months [24, 25]. Underlying diseases have been reported as the risk factors for IPD [21, 26], and reported underlying diseases including myocardial infarction, cerebrovascular disease, diabetes mellitus, malignancy, and so on. Our findings showed that 75 (59%) patients had some form of underlying diseases. The most common underlying conditions were malignancy (11%), pneumonia (11%), hypertension (9.4%) and cirrhosis (7.9%). In parallel with previous studies, cancer patients are especially susceptible to severe pneumococcal infections, because of their weakened immune systems [27, 28]. Cirrhosis is a well-known predisposing factor for IPD and cirrhosis affected 7.9% of the patients in our study. A multicenter study reported that cirrhosis increased the risk of death from IPD more than any other condition analyzed [29, 30]. The reasons for this observation have not been elucidated. Propst–Graham et al. [31] showed that increased mortality from pneumococcal pneumonia in cirrhotic rats is related to impairments in both preneutrophil-mediated and later neutrophil-mediated innate pulmonary killing of the *S. pneumoniae*. Moreover, the incidence of underlying diseases was significantly higher in the adult group than in the pediatric group. This can be explained by the fact that adult patients with underlying diseases have weak immune systems. Hence, researchers hypothesized that control of underlying diseases is a measure to decrease cases of pneumococcal infections [32, 33]. Of the 24 (47.1%) pediatric patients with underlying diseases, the most common conditions were pneumonia and anemia. This finding is inconsistent with previous studies which reported that the main underlying disease was leukemia for children with IPD [16, 34]. The underlying disease rate of children in our study was higher than in other studies in China [16, 35]. While in 67.1% of adult patients who had underlying diseases, hypertension was most frequent, followed by malignancy and cirrhosis. Tsuchiya et al. reported a similar result that 15.1% of adult IPD patients had liver diseases [36].

In the present study, presenting symptoms mainly included fever, cough, vomiting, consciousness obstacle, and headache. Among all the symptoms, fever, cough, and seizures were significantly different in the two age groups, occurring more commonly in pediatric patients. This finding is in line with several previous studies in which fever was present in almost all cases of pneumococcal meningitis [37, 38]. Another study has shown that a delay in the initiation of therapy introduces the potential for increased morbidity and mortality of community-acquired bacterial meningitis [39]. Thus, these findings may suggest that patients presenting with fever and another mental status should be given empirical antibiotic therapy before the blood or CSF culture results were reported.

Elevated blood WBC count and neutrophil percentage are traditionally reported to be a reliable indicator of bacterial infection. In the present study, blood WBC was elevated in 60% of pediatric cases and 52% of adult cases, respectively. The neutrophil percentage was elevated in 46% of pediatric cases and 80% of adult cases. Nevertheless, it is important to highlight that 40% and 54% of the children, and 40% and 54% of the adults did not have elevated blood WBC counts and neutrophil percentages, suggesting that more laboratory tests should be combined to help diagnose IPD before *S. pneumoniae* identification. Bacterial meningitis is characterized by high levels of CSF WBC counts and CSF protein and decreased levels of CSF glucose [40]. Forty-nine patients with pneumococcal meningitis included in this study had routine CSF analysis. Overall, 95.9%, 93.9%, and 83.7% of patients had elevated levels of WBC counts and CSF protein and decreased levels of glucose, respectively. Inconsistent with our result, Wang et al. [38] reported that 11.5% of the children had normal CSF cell counts. These results emphasize the importance of a comprehensive analysis of CSF cell counts, CSF protein, and glucose levels. Additionally, WBC count, neutrophil percentage and protein level in the CSF samples were significantly higher in the pediatric group than in the adult group. This finding may be explained that adults mount stronger immune responses than children to prevent or limit infection.

Since the clinical resistance to penicillin in *S. pneumoniae* was first reported in 1965 [41], antibiotic resistance among *S. pneumoniae* continues to increase and become a severe problem worldwide [42]. The high resistance rates for penicillin have been reported in many countries, such as the United States, Tunisia, France, and Spain [43–45]. A previous study reported that the resistance trend of *S. pneumoniae* was regionally different [46]. The nonsusceptibility rates of penicillin in bacteremia and meningitis isolates (1.3% and 65.9%) were lower than in a study conducted in Beijing China (8.5% and 71.9%) [16]. Consistent with many studies [47–49], *S. pneumoniae* in the present study is generally sensitive to vancomycin, linezolid, and levofloxacin, with 100% susceptibility. Erythromycin, clarithromycin, and azithromycin are the three most widely used macrolide antibiotics in the clinic. In the current study, the high resistance rates to macrolide ranged from 88.9% to 91.9%, which were either similar to or higher than those reported in previous studies. A study from the Asian Network for Surveillance of Resistant Pathogens (ANSORP) revealed that resistance rates to macrolides in pneumococcal isolates ranged from 64.7% to 67.2% in Asian countries [50]. The resistance rates of erythromycin, clindamycin, azithromycin, and tetracycline were 92%, 81.33%, and 90.67% in Shanghai, China [51]. Due to the high resistance to macrolide, the clinical utility of macrolide against infections caused by *S. pneumoniae* is low.

In this study, antimicrobial treatment was frequently initiated with 3-generation cephalosporin (44.88%), followed by carbapenems, vancomycin, and latamoxef. Seventy-four patients had changed antibiotics according to the susceptibility of the pathogen. Vancomycin, meropenem, and ceftriaxone were the most common antibiotics. In our study, the total mortality was 5.5%. This rate is lower than those described in other studies, showing a higher 30-day mortality rate, ranging from 16%–46.2% [37, 38, 52, 53]. This may be because 29.9% of patients deteriorated in the hospital and gave up treatment, but we did not follow up with patients and observe the 30-day mortality rate.

Our study has some limitations. Firstly, it was performed with data from only one hospital with a limited number of patients in adult and pediatric groups. Hence, data from this study may not reflect the clinical characteristics of IPD in the NHAR. Due to the nature of the retrospective study, we could not confirm the information on the vaccination history of individuals and identify the capsule type of *S. pneumoniae* isolates. Similarly, we could not observe a 30-day mortality rate, and this may result in the mortality rate in this study being lower than in other studies.

## 5. Conclusion

In conclusion, IPD is a serious infectious disease that is still endemic in the NHAR. Our results showed that IPD can occur at any age, especially the children and adults with underlying diseases. The clinical characteristics of IPD varied with age.

The antibiotic resistance rates are of serious concern in patients with pneumococcal meningitis, and this emphasizes the need for rapid initiation of using appropriate empiric antibiotic therapy if meningitis is suspected. These results may provide a reference basis for the diagnosis and empiric treatment of IPD in the region. However, due to a limited number of patients from only one hospital, large-scale multicenter surveillance of IPD should be implemented in the future.

## Figures and Tables

**Table 1:** Comparison of demographic and clinical characteristics between pediatric and adult IPD cases in Ningxia Hui Autonomous Region, China, 2013–2021.

Characteristic	Age groups		*P* value
	≤16 years (*N* = 51), *N* (%)	>16 years (*N* = 76), *N* (%)	
Gender			
Male	27 (52.9)	45 (59.2)	0.584
Female	24 (47.1)	31 (40.8)	
Underlying disease	24 (47.1)	51 (67.1)	0.028
Pneumonia	10 (19.6)	4 (5.3)	0.019
Malignancy	3 (5.9)	11 (14.5)	0.157
Cirrhosis	0 (0)	10 (13.2)	0.006
Hypertension	0 (0)	12 (15.8)	0.002
Anemia	6 (11.76)	2 (2.63)	0.059
Brain trauma	2 (3.9)	3 (4)	>0.9999
Symptoms at presentation			
Fever	48 (94.1)	56 (73.7)	0.004
Cough	21 (41.2)	13 (17.1)	0.004
Vomiting	13 (25.5)	12 (15.8)	0.255
Consciousness obstacle	9 (17.7)	15 (19.7)	0.821
Headache	5 (9.8)	17 (22.4)	0.094
Sputum	7 (13.7)	12 (15.8)	0.805
Seizures	12 (23.5)	3 (4.0)	0.001

**Table 2:** Comparison of laboratory analysis of blood and CSF between pediatric and adult IPD cases in Ningxia Hui autonomous region, China, 2013–2021.

Laboratory information	Age groups		*P* value
	≤16 years	>16 years	
Peripheral blood			
White blood cell (WBC) increasing, *N* (%)	30 (60)	39 (52)	0.463
Neutrophil percentage increasing, *N* (%)	23 (46)	60 (80)	<0.001
Cerebral spinal fluid			
White blood cell (/mm^3^), (median)	81	3840	<0.001
Neutrophil (%), (median)	61.5	82	0.012
Lymphocyte (%), (median)	9	7	0.744
Protein level (g/L), (median)	2.135	4.99	0.019
Cl^−^ (mmol/L), (median)	115	116	0.276
Glucose (mmol/L), (median)	1.1	1.1	0.557
PCT (ng/ml), *N*			
<0.5	7	12	0.998
0.5 ≤ PCT < 2	9	14	
2 ≤ PCT < 10	7	12	
≥10	13	22	

**Table 3:** Susceptibilities of *S. pneumoniae* isolates to antimicrobial agents.

Antibiotics name	R (%)	I (%)	S (%)
Penicillin G (nonmeningitis)	0	1.3	98.7
Penicillin G (meningitis)	65.9	0	34.1
Ceftriaxone (nonmeningitis)	3.2	4.8	91.9
Ceftriaxone (meningitis)	4.5	36.4	59.1
Cefotaxime (nonmeningitis)	5	7.5	87.5
Cefotaxime (meningitis)	8.3	41.7	50
Cefuroxime	28.9	8.9	62.2
Meropenem	5.8	19.2	75
Levofloxacin	0	0	100
Trimethoprim/sulfamethoxazole	67.4	9.5	23.1
Clindamycin	91.9	1	7.1
Azithromycin	88.9	0	11.1
Erythromycin	91.3	4	4.8
Linezolid	0	0	100
Vancomycin	0	0	100
Chloramphenicol	14	0	86
Tetracycline	95.1	1.2	3.7

S: sensitive; I: intermediate; R: resistant.

## Data Availability

The data in this study are available from the corresponding author upon reasonable request.

## References

[1] Weiser J. N., Ferreira D. M., Paton J. C. (2018). Streptococcus pneumoniae: transmission, colonization and invasion. *Nature Reviews Microbiology*.

[2] Dunn M. G., Lessa F. C., Sanchez J. (2021). Impact of 13-valent pneumococcal conjugate vaccine on nasopharyngeal carriage rates of Streptococcus pneumoniae in a rural community in the Dominican republic. *The Journal of Infectious Diseases*.

[3] Deng X., Church D., Vanderkooi O. G., Low D. E., Pillai D. R. (2013). Streptococcus pneumoniae infection: a Canadian perspective. *Expert Review of Anti-infective Therapy*.

[4] De Ste Croix M., Mitsi E., Morozov A. (2020). Phase variation in pneumococcal populations during carriage in the human nasopharynx. *Scientific Reports*.

[5] Marrie T. J., Majumdar S. R., Eurich D. (2017). Invasive Pneumococcal Disease: Still Lots to Learn and a Need for Standardized Data Collection Instruments. *Canadian Respiratory Journal*.

[6] Engholm D. H., Kilian M., Goodsell D. S., Andersen E. S., Kjærgaard R. S. (2017). A visual review of the human pathogen Streptococcus pneumoniae. *FEMS Microbiology Reviews*.

[7] Coronas E., Martinot A., Varon E., Wallet F., Dubos F. (2021). Stable incidence of invasive pneumococcal disease in children in northern France from 2014 through 2018. *The Pediatric Infectious Disease Journal*.

[8] Marrie T. J., Tyrrell G. J., Majumdar S. R., Eurich D. T. (2018). Effect of age on the manifestations and outcomes of invasive pneumococcal disease in adults. *The American Journal of Medicine*.

[9] Nakamura T., Cohen A. L., Schwartz S. (2021). The global landscape of pediatric bacterial meningitis data reported to the world health organization-coordinated invasive bacterial vaccine-preventable disease surveillance Network, 2014-2019. *The Journal of Infectious Diseases*.

[10] Jayaraman R., Varghese R., Kumar J. L. (2019). Invasive pneumococcal disease in Indian adults: 11 years’ experience. *Journal of Microbiology, Immunology, and Infection*.

[11] Wahl B., O’Brien K. L., Greenbaum A. (2018). Burden of Streptococcus pneumoniae and Haemophilus influenzae type b disease in children in the era of conjugate vaccines: global, regional, and national estimates for 2000-15. *Lancet Global Health*.

[12] Chen H., Matsumoto H., Horita N., Hara Y., Kobayashi N., Kaneko T. (2021). Prognostic factors for mortality in invasive pneumococcal disease in adult: a system review and meta-analysis. *Scientific Reports*.

[13] Vasoo S., Singh K., Chow C., Lin R. T. P., Hsu L. Y., Tambyah P. A. (2010). Pneumococcal carriage and resistance in children attending day care centers in Singapore in an early era of PCV-7 uptake. *Journal of Infection*.

[14] Zhao C., Xie Y., Zhang F. (2020). Investigation of Antibiotic Resistance, Serotype Distribution, and Genetic Characteristics of 164 Invasive Streptococcus pneumoniae from North China between April 2016 and October 2017. *Infection and Drug Resistance*.

[15] Zhou M., Wang L., Wang Z. (2022). Molecular characterization of penicillin-binding Protein2x, 2b and 1a of Streptococcus pneumoniae causing invasive pneumococcal diseases in China: a multicenter study. *Frontiers in Microbiology*.

[16] Jiang M., Wang X., Zhu L. (2022). Clinical characteristics, antimicrobial resistance, and risk factors for mortality in paediatric invasive pneumococcal disease in Beijing, 2012-2017. *BMC Infectious Diseases*.

[17] CLSI Performance Standards for Antimicrobial Susceptibility Testing (2020). *CLSI Supplement M100*.

[18] von Mollendorf C., Tempia S., von Gottberg A. (2017). Estimated severe pneumococcal disease cases and deaths before and after pneumococcal conjugate vaccine introduction in children younger than 5 years of age in South Africa. *PLoS One*.

[19] Minami M., Sakakibara R., Imura T., Morita H., Kanemaki N., Ohta M. (2014). Prevalence and antimicrobial susceptibility pattern of Streptococcus pneumoniae at general hospital in the central region of Japan from december 2013 to february 2014. *Journal of Biosciences and Medicines*.

[20] Geng Q., Zhang T., Ding Y. (2014). Molecular characterization and antimicrobial susceptibility of Streptococcus pneumoniae isolated from children hospitalized with respiratory infections in Suzhou, China. *PLoS One*.

[21] Chan T., Tay M. Z., Kyaw W. M., Chow A., Ho H. J. (2020). Epidemiology, vaccine effectiveness, and risk factors for mortality for pneumococcal disease among hospitalised adults in Singapore: a case-control study. *BMC Infectious Diseases*.

[22] Al Musawi M. (2012). A retrospective epidemiological study of invasive pneumococcal infections in children aged 0-5 years in Bahrain from 1 January 1999 to 31 December 2003. *Vaccine*.

[23] Zhao W., Pan F., Wang B. (2019). Epidemiology characteristics of Streptococcus pneumoniae from children with pneumonia in Shanghai: a retrospective study. *Frontiers in Cellular and Infection Microbiology*.

[24] Sharma P. K., Ramakrishnan R., Hutin Y. (2009). Scrub typhus in Darjeeling, India: opportunities for simple, practical prevention measures. *Transactions of the Royal Society of Tropical Medicine and Hygiene*.

[25] Chapagain R. H., Agrawal S., Pokharel S. (2020). Clinico-laboratory profile, complications and therapeutic outcome of scrub typhus in children. *Journal of Nepal Health Research*.

[26] Imai K., Petigara T., Kohn M. A. (2018). Risk of pneumococcal diseases in adults with underlying medical conditions: a retrospective, cohort study using two Japanese healthcare databases. *BMJ Open*.

[27] Garcia-Vidal C., Ardanuy C., Gudiol C. (2012). Clinical and microbiological epidemiology of Streptococcus pneumoniae bacteremia in cancer patients. *Journal of Infection*.

[28] Hupf H. B., Beaver J. E. (1970). Cyclotron production of carrier-freegallium-67. *The International Journal of Applied Radiation and Isotopes*.

[29] Feikin D. R., Schuchat A., Kolczak M. (2000). Mortality from invasive pneumococcal pneumonia in the era of antibiotic resistance, 1995-1997. *American Journal of Public Health*.

[30] Choi S. H., Park H. G., Jun J. B. (2009). Clinical characteristics and outcomes of pneumococcal bacteremia in adult patients with liver cirrhosis. *Diagnostic Microbiology and Infectious Disease*.

[31] Propst-Graham K. L., Preheim L. C., Vander Top E. A., Snitily M. U., Gentry-Nielsen M. J. (2007). Cirrhosis-induced defects in innate pulmonary defenses against Streptococcus pneumoniae. *BMC Microbiology*.

[32] Kyaw M. H., Rose,  Jr. C., Fry A. (2005). The influence of chronic illnesses on the incidence of invasive pneumococcal disease in adults. *The Journal of Infectious Diseases*.

[33] Shigayeva A., Rudnick W., Green K. (2016). Invasive pneumococcal disease among immunocompromised persons: implications for vaccination programs. *Clinical Infectious Diseases*.

[34] Kaplan S. L., Barson W. J., Lin P. L. (2019). Invasive pneumococcal disease in children’s hospitals: 2014-2017. *Pediatrics*.

[35] Cai K., Wang Y., Guo Z., Xu X., Li H., Zhang Q. (2018). Clinical characteristics and antimicrobial resistance of pneumococcal isolates of pediatric invasive pneumococcal disease in China. *Infection and Drug Resistance*.

[36] Tsuchiya M., Miyazaki H., Takata M. (2022). Comparative characteristics of the background and blood test findings in adults with pneumococcal pneumonia and invasive pneumococcal disease: a retrospective study. *Journal of Infection and Chemotherapy*.

[37] Ostergaard C., Konradsen H. B., Samuelsson S. (2005). Clinical presentation and prognostic factors of Streptococcus pneumoniae meningitis according to the focus of infection. *BMC Infectious Diseases*.

[38] Wang W., Han H., Du L., Li Z., Wu Y. (2022). Clinical features and outcomes of Streptococcus pneumoniae meningitis in children: a retrospective analysis of 26 cases in China. *Neuropediatrics*.

[39] Aronin S. I., Peduzzi P., Quagliarello V. J. (1998). Community-acquired bacterial meningitis: risk stratification for adverse clinical outcome and effect of antibiotic timing. *Annals of Internal Medicine*.

[40] Seehusen D. A., Reeves M. M., Fomin D. A. (2003). Cerebrospinal fluid analysis. *American Family Physician*.

[41] Kislak J. W., Daly A. K., Finland M., Lawrence M. B. R. (1965). Susceptibility of pneumococci to nine antibiotics. *The American Journal of the Medical Sciences*.

[42] Wang C. Y., Chen Y. H., Fang C. (2019). Antibiotic resistance profiles and multidrug resistance atterns of Streptococcus pneumoniae in pediatrics: a multicenter retrospective study in mainland China. *Medicine (Baltimore)*.

[43] Whitney C. G., Farley M. M., Hadler J. (2000). Increasing prevalence of multidrug-resistant Streptococcus pneumoniae in the United States. *New England Journal of Medicine*.

[44] Raddaoui A., Simoes A. S., Baaboura R. (2015). Serotype distribution, antibiotic resistance and clonality of Streptococcus pneumoniae isolated from immunocompromised patients in Tunisia. *PLoS One*.

[45] Adam D. (2002). Global antibiotic resistance in Streptococcus pneumoniae. *Journal of Antimicrobial Chemotherapy*.

[46] Song J. H., Lee N., Ichiyama S. (1999). Spread of drug-resistant Streptococcus pneumoniae in asian countries: asian Network for surveillance of resistant pathogens (ANSORP) study. *Clinical Infectious Diseases*.

[47] Zhang J., Hu D. K., Gao C. Y. (2020). Homology analysis of 51 penicillin-intermediate Streptococcus pneumoniae isolates from Wenzhou City, China. *Journal of International Medical Research*.

[48] Sader H. S., Mendes R. E., Le J., Denys G., Flamm R. K., Jones R. N. (2019). Antimicrobial susceptibility of Streptococcus pneumoniae from north America, europe, Latin America, and the asia-pacific region: results from 20 Years of the SENTRY antimicrobial surveillance program (1997-2016). *Open Forum Infectious Diseases*.

[49] Chen K. J., Chong Y. J., Sun M. H. (2021). Streptococcus pneumoniae endophthalmitis: clinical settings, antibiotic susceptibility, and visual outcomes. *Scientific Reports*.

[50] Kim S. H., Chung D. R., Song J. H. (2020). Changes in serotype distribution and antimicrobial resistance of Streptococcus pneumoniae isolates from adult patients in Asia: emergence of drug-resistantnon-vaccine serotypes. *Vaccine*.

[51] Li X. X., Xiao S. Z., Gu F. F. (2019). Serotype distribution, antimicrobial susceptibility, and multilocus sequencing type (MLST) of Streptococcus pneumoniae from adults of three hospitals in Shanghai, China. *Frontiers in Cellular and Infection Microbiology*.

[52] Christensen J. S., Jensen T. G., Kolmos H. J., Pedersen C., Lassen A. (2012). Bacteremia with Streptococcus pneumoniae: sepsis and other risk factors for 30-day mortality--a hospital-based cohort study. *European Journal of Clinical Microbiology & Infectious Diseases*.

[53] Skjold-Odegaard B., Hamid S., Lindeman R. J., Ersdal H. L., Soreide K. (2021). Deciphering the inflection points to achieve proficiency for each procedure step during training in laparoscopic appendicectomy. *BJS Open*.

